# RISING STARS: Evidence for established and emerging forms of β-cell death

**DOI:** 10.1530/JOE-23-0378

**Published:** 2024-07-04

**Authors:** Kaitlyn A Colglazier, Noyonika Mukherjee, Christopher J Contreras, Andrew T Templin

**Affiliations:** 1 Lilly Diabetes Center of Excellence, Indiana Biosciences Research Institute, Indianapolis, Indiana, USA; 2 Department of Biochemistry & Molecular Biology, Indiana University School of Medicine, Indianapolis, Indiana, USA; 3 Division of Endocrinology, Department of Medicine, Roudebush VA Medical Center and Indiana University School of Medicine, Indianapolis, Indiana, USA; 4 Center for Diabetes and Metabolic Diseases, Indiana University School of Medicine, Indianapolis, Indiana, USA

**Keywords:** diabetes, β-cell death, inflammation, necroptosis, ferroptosis, pyroptosis

## Abstract

β-Cell death contributes to β-cell loss and insulin insufficiency in type 1 diabetes (T1D), and this β-cell demise has been attributed to apoptosis and necrosis. Apoptosis has been viewed as the lone form of programmed β-cell death, and evidence indicates that β-cells also undergo necrosis, regarded as an unregulated or accidental form of cell demise. More recently, studies in non-islet cell types have identified and characterized novel forms of cell death that are biochemically and morphologically distinct from apoptosis and necrosis. Several of these mechanisms of cell death have been categorized as forms of regulated necrosis and linked to inflammation and disease pathogenesis. In this review, we revisit discoveries of β-cell death in humans with diabetes and describe studies characterizing β-cell apoptosis and necrosis. We explore literature on mechanisms of regulated necrosis including necroptosis, ferroptosis and pyroptosis, review emerging literature on the significance of these mechanisms in β-cells, and discuss experimental approaches to differentiate between various mechanisms of β-cell death. Our review of the literature leads us to conclude that more detailed experimental characterization of the mechanisms of β-cell death is warranted, along with studies to better understand the impact of various forms of β-cell demise on islet inflammation and β-cell autoimmunity in pathophysiologically relevant models. Such studies will provide insight into the mechanisms of β-cell loss in T1D and may shed light on new therapeutic approaches to protect β-cells in this disease.

## Invited Author’s profile


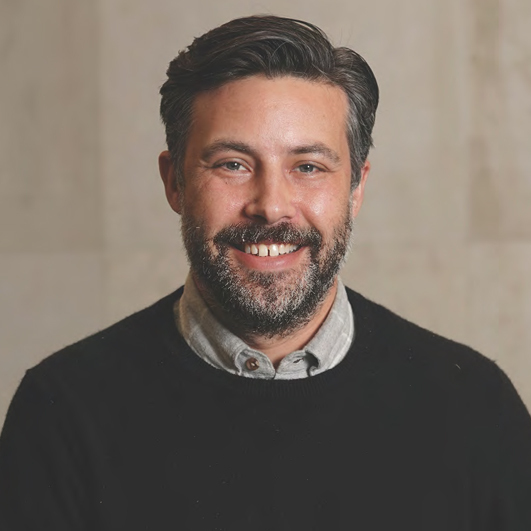


**Andrew T Templin**, PhD, is a β-cell biologist and diabetologist who holds positions at Indiana University School of Medicine, Indiana Biosciences Research Institute, and the Roudebush VA Medical Center. Research in the Templin Lab focuses on understanding the relationship between islet immune responses and β-cell dysfunction and death in the setting of both major forms of diabetes. Emphasis is placed on the concept that β-cell intrinsic properties are drivers of islet inflammation and immune responses, and together these promote a system of β-cell dysfunction and loss that leads to diabetes. In this context, the Templin Lab investigates underappreciated mechanisms of islet inflammation and β-cell cytotoxicity, and the relationship of these to diabetogenic β-cell loss. Through this work, the Templin Lab aims to advance our understanding of β-cell demise and improve the health of individuals with diabetes. Andrew is a native Hoosier who enjoys travel, soccer, the Chicago Cubs, Golden Retrievers, and spending time with family.

## Introduction

β-Cell death contributes to β-cell loss, insufficient insulin secretion, and hyperglycemia in the pathogenesis of type 1 diabetes (T1D) (Mandrup-Poulsen 2001, Mathis *et al.* 2001, Cnop *et al.* 2005, Rhodes 2005), and studies of the cellular and molecular mechanisms that mediate β-cell death have focused principally on β-cell apoptosis (Augstein *et al.* 1998, Mandrup-Poulsen 2001, Butler *et al.* 2003, 2007) and necrosis (Hoorens *et al.* 2001, Scarim *et al.* 2001, Fehsel *et al.* 2003). Apoptosis is triggered by intrinsic or extrinsic signals and has classically been considered the lone form of programmed cell death (PCD) (Alberts *et al.* 2002), whereas necrosis has been regarded as an unprogrammed or accidental form of cell death that results from chemical or physical injury (Buja *et al.* 1993, Majno & Joris 1995). More recent studies in non-islet cell types have identified and characterized several additional mechanisms of PCD (Proskuryakov *et al.* 2003, Berghe *et al.* 2014, Conrad *et al.* 2016) that are molecularly, morphologically, and functionally distinct from apoptosis (Linkermann & Green 2014, Man *et al.* 2017, Zheng & Conrad 2020, Bertheloot *et al.* 2021). Although there is growing evidence that β-cells are susceptible to these emerging forms of PCD (Bruni *et al.* 2018, Yang *et al.* 2020, Tonnus *et al.* 2021, Contreras *et al.* 2022), the roles of these forms of β-cell demise in diabetogenic β-cell loss have not been well established. In this review, we discuss our current understanding of β-cell death in human diabetes, review literature on mechanisms of regulated necrosis in non-islet cell types, examine the relevance of emerging forms of cell death to diabetogenic β-cell loss, explore heterogeneity of β-cell death responses, and describe experimental approaches to differentiate between various mechanisms of cell death. We begin by examining evidence for β-cell death as a factor in the pathogenesis of human T1D.

### β-Cell death in the pathogenesis of human T1D

Interest in understanding and targeting mechanisms of β-cell death to combat T1D arises from the extensive literature linking β-cell cytotoxicity to insulin insufficiency and hyperglycemia in this disease. Although the decreased abundance of insulin-producing β-cells was observed in T1D as early as the 1950s (Maclean & Ogilvie 1959), such observations were not linked to increased rates of β-cell death until decades later. Methods developed in the 1990s facilitated the detection and quantification of dead cells in tissue sections, including terminal deoxy-nucleotidyl transferase dUTP nick end labeling (TUNEL) (Gorczyca *et al.* 1992), annexin V staining (Vermes *et al.* 1995), and propidium iodide staining (Douglas *et al.* 1995). Utilizing these techniques, studies were conducted to quantify β-cell death in human pancreas sections from non-diabetic and T1D organ donors, and evidence emerged that β-cell death is elevated in individuals with T1D. For example, two early studies across 23 non-diabetic and 51 T1D pancreas samples revealed that β-cell death was increased approximately two-fold in those with T1D (Meier *et al.* 2005, Butler *et al.* 2007). Although these studies observed that increased rates of β-cell death contribute to β-cell loss and insulin insufficiency in individuals with T1D, they also provoked several questions and led to additional work to better understand the role of β-cell death in T1D disease pathogenesis.

Several factors must be considered when evaluating β-cell death in human pancreas sections and interpreting relevance to T1D disease pathogenesis. One important consideration is that human pancreas samples are typically donated years or decades after diagnosis with T1D. Thus, the rate of β-cell death quantified in these samples probably underestimates that which occurs during the period of active β-cell demise, which is thought to begin years prior to the clinical onset of the disease. Substantial loss of β-cell mass at the time of T1D diagnosis likely reduces the potential to observe ongoing β-cell death, and remaining β-cells may be those least susceptible to cytotoxic insults (Rui *et al.* 2017). Indeed, pancreas samples from organ donors with T1D exhibit only modestly increased rates of β-cell death, with studies typically finding ~1 dead β-cell in every two to three diabetic islets (Meier *et al.* 2005, Butler *et al.* 2007), and it has been debated whether this low rate of β-cell death could account for the diminished β-cell mass observed in T1D. We feel it is essential to consider the timing of active β-cell demise in T1D disease pathogenesis and the fact that dead islet cells exist for only a finite period of time before being cleared by islet macrophages *in situ*. Thus, establishing the true contribution of β-cell death to β-cell loss in T1D requires both capturing the active period of β-cell demise and determining how accumulation of cell death translates to diminished β-cell mass over time. Given that human pancreas tissue is collected at a single time point (post mortem), our ability to determine the contribution of β-cell death to β-cell loss in human T1D pancreas samples is limited.

### Biomarkers of β-cell death in human T1D

To address this limitation, studies have aimed to identify and measure biomarkers of β-cell death in living humans earlier in disease progression. For example, methodologies have emerged that facilitate the analysis of β-cell death *in situ* using the quantification of circulating unmethylated insulin or amylin promoter DNA from blood samples. These particular types of DNA are found exclusively in β-cells (Akirav *et al.* 2011, Lehmann-Werman *et al.* 2016, Olsen *et al.* 2016) and their release into circulation provides specific biomarkers of β-cell death *in vivo*. Using this approach, studies determined that unmethylated insulin and amylin DNA are increased in serum from individuals with newly diagnosed T1D and that it remains elevated eight weeks after disease diagnosis (Akirav *et al.* 2011, Fisher *et al.* 2013, Olsen *et al.* 2016, Neyman *et al.* 2019). Other work failed to identify increased unmethylated insulin DNA in those with recent onset T1D (Neiman *et al.* 2020), suggesting that heterogeneity in the timeline (relapsing-remitting) or mode of active β-cell demise may exist in late T1D pathogenesis. The use of this technique also revealed that unmethylated insulin DNA is elevated following islet transplantation, indicative of β-cell death in this setting (Husseiny *et al.* 2014, Neiman *et al.* 2020). Although additional work is needed to understand β-cell death in early T1D pathogenesis, these studies reinforce findings from pancreas sections that show increased rates of β-cell death in T1D and support a model wherein β-cell death contributes to β-cell loss, insulin insufficiency, and hyperglycemia in this disease.

Approaches to identify biomarkers of β-cell demise early in T1D pathogenesis may also provide great clinical and therapeutic value. Such biomarkers could be used to identify individuals at risk for T1D and allow for initiation of β-cell protective therapies at a time when significant β-cell mass still exists. In this context, the presence of islet autoantibodies including insulin (IAA), glutamic acid decarboxylase (GAD), protein tyrosine phosphatase (IA2), and zinc transporter 8 (ZnT8) are being used as biomarkers for T1D disease progression (Ziegler *et al.* 2013). In a study of non-diabetic individuals positive for at least 2 islet autoantibodies, a 14-day treatment with an anti-CD3 monoclonal antibody significantly prolonged the time to T1D onset (Sims *et al.* 2021), highlighting the therapeutic utility of early detection of β-cell demise. In addition to islet autoantibodies, other biomarkers of β-cell failure including unmethylated insulin and amylin promoter DNA, proinsulin:C-peptide ratio, and miRNAs (miR375, miR21, miR34a, miR146a) are being investigated toward the development of reliable biomarker panels for the detection of β-cell demise in early T1D (Roggli *et al.* 2010, Tersey *et al.* 2012, Erener *et al.* 2013, Mirmira *et al.* 2016). Moreover, techniques to quantify cell mass and cell death *in vivo* are emerging (Cordeiro *et al.* 2010, Khanna *et al.* 2010, Yamazaki *et al.* 2020, Lehrstrand *et al.* 2024), and future studies might apply these *in vivo* imaging methodologies to better understand the timing and extent of β-cell death in early T1D.

## Mechanisms of β-cell death

Given the current limitations of studying β-cell death in human disease, much of our understanding of β-cell demise in T1D is derived from experimental models. Numerous *in vitro* and* in vivo* studies have shed light on the occurrence and mechanisms of β-cell death in T1D (as reviewed here), as well as intrinsic properties of β-cells (such as high metabolic demand, high secretory demand, and limited antioxidant capacity) that make them susceptible to cytotoxic insults (Grankvist *et al.* 1981, Kulkarni *et al.* 2022). Many β-cell cytotoxic stimuli have been identified, including proinflammatory cytokines, autoimmunity, glucotoxicity, oxidative stress, and ER stress. Here, we focus on the various cell-intrinsic mechanisms of β-cell death that are activated in response to such stimuli. With respect to β-cell-intrinsic death pathways, most work has focused on apoptosis and necrosis, which we categorize as established mechanisms of β-cell death herein. In addition, several mechanisms of programmed cell death (PCD) that are distinct from apoptosis and necrosis have emerged in the last two decades, but the significance of these forms of cell demise to diabetogenic β-cell death is unclear. In this section, we examine the literature that underlies our current understanding of established forms of cell death such as apoptosis and necrosis, as well as emerging forms of cell death such as necroptosis, ferroptosis, and pyroptosis. We also explore literature that suggests these types of cell death are germane to β-cells and discuss considerations for accurate experimental characterization of various forms of cell death.

### Established mechanisms of β-cell death

#### Apoptosis

Apoptosis is the most well-known and well-characterized form of PCD (see Table 1 for a list of abbreviations). It is tightly regulated by cell-intrinsic and cell-extrinsic signaling mechanisms (Elmore 2007), and it is required for several physiological processes including development, tumor suppression, and removal of damaged or unwanted cells during normal cellular turnover (Kerr *et al.* 1972, Vaux & Korsmeyer 1999, Norbury & Hickson 2001). Apoptotic programs are critically dependent on caspases, a family of cysteine-aspartic proteases that are activated in response to specific stimuli (Chen & Wang 2002). With respect to apoptosis signaling, caspases can be categorized as initiator caspases (caspase 2, 8, 9, and 10) or executioner caspases (caspase 3, 6, and 7) (Cohen 1997, Chen & Wang 2002). Upon appropriate stimulation, initiator caspases are activated by autoproteolysis; these initiator caspases activate executioner caspases via proteolysis, and executioner caspases then initiate apoptotic programs via proteolysis of key structural, cell cycle, and signal transduction proteins (Parrish *et al.* 2013). Cells undergoing apoptosis exhibit cytoplasmic shrinkage, chromatin condensation, DNA fragmentation, and plasma membrane blebbing (Elmore 2007). These membrane blebs encompass cellular contents to form apoptotic bodies that are then taken up by phagocytes through efferocytosis; this feature of apoptosis is critical in that it allows apoptotic cells to be removed in a non-inflammatory manner (Boada-Romero *et al.* 2020).

**Table 1:** Cell death-related abbreviations.

Abbreviation^a^	Expanded form
PCD	Programmed cell death
T1D	Type 1 diabetes
T2D	Type 2 diabetes
TUNEL	Terminal deoxynucleotidyl transferase dUTP nick end labeling
STZ	Streptozotocin
HFD	High fat diet
IL	Interleukin
TNFα	Tumor necrosis factor alpha
TNFR	Tumor necrosis factor receptor
IFNγ	Interferon gamma
RIPK1	Receptor interacting protein kinase 1
RIPK3	Receptor interacting protein kinase 3
MLKL	Mixed lineage kinase domain-like pseudokinase
DAMPs	Danger-associated molecular patterns
PAMPs	Pathogen-associated molecular patterns
TRADD	Tumor necrosis factor receptor type 1-associated death domain
cIAP1/2	Cellular inhibitors of apoptosis proteins
LUBAC	Linear ubiquitin chain assembly complex
IFNGR	Interferon gamma receptor
TLR	Toll-like receptors
TRAILR	TNF-related apoptosis-inducing ligand receptor
RHIM	RIP homotypic interaction motif
Nec-1	Necrostatin-1
NFκB	Nuclear factor κB
GSH	Glutathione
GPX4	Glutathione peroxidase 4
NADPH	Nicotinamide adenine dinucleotide phosphate
ROS	Reactive oxygen species
LDH	Lactate dehydrogenase
Fer-1	Ferrostatin
GSDM	Gasdermin
HMGB1	High mobility group box 1
NLRP3	NLR family pyrin domain containing 3
PRR	Pattern-recognition receptors
NLR	NOD-like receptor
Poly I:C	Polyinosinic:polycytidylic acid

^a^Key abbreviations mentioned within the review in the order mentioned.

There is a great deal of evidence that β-cells undergo apoptosis in response to diabetes-relevant cell death stimuli (Mandrup-Poulsen 2001, Hui *et al.* 2004, Thomas *et al.* 2009). Studies performed by Yamada *et al.* in 1999 found that Fas signaling in β-cells results in cell death characterized by chromatin condensation, nucleolar disintegration, DNA fragmentation, and annexin V positivity, hallmarks of apoptosis (Yamada *et al.* 1999). In 2005, Liadis and colleagues found that caspase 3-deficient mice were protected from hyperglycemia following multiple low-dose streptozotocin (STZ) treatment, and this was related to reduced β-cell loss (Liadis *et al.* 2005). Similarly, a study utilizing caspase 3-deficient islets or a small molecule caspase 3 inhibitor found that caspase 3 activity mediates amyloid-induced β-cell death (Li *et al.* 2008). Studies of proinflammatory cytokine-induced β-cell death have also identified apoptosis as a key mediator of β-cell cytotoxicity, with studies finding that a cytokine cocktail (IL-1β + IFNγ + TNFα) elicits β-cell death in association with caspase 3 activation (Grunnet *et al.* 2009), and that suppressor of cytokine signaling-1 (SOCS-1) mediates IL-1β + IFNγ + TNFα-induced β-cell death in a caspase 3-dependent fashion (Zaitseva *et al.* 2009). In a study of β-cell-specific loss of caspase 8, islets were protected from Fas ligand and ceramide-induced cell death *in vitro*, and mice were protected from STZ-induced hyperglycemia *in vivo* (Liadis *et al.* 2007). Notably, this study also showed that the loss of caspase 8 in β-cells results in elevated rates of islet cell death and glucose intolerance in aged chow-fed mice *in vivo*, indicating distinct roles for β-cell caspase 8 under these conditions (Liadis *et al.* 2007).

Apoptosis has also been identified as a key contributor to β-cell demise in the context of islet transplantation (Emamaullee *et al.* 2007, McCall & Shapiro 2012, Pepper *et al.* 2017). For example, treatment of isolated mouse islets with a small molecule pan-caspase inhibitor (zVAD) prior to transplantation combined with 5 days of zVAD treatment after transplant resulted in significantly improved rates of euglycemia following islet transplantation, and the glycemic benefit of zVAD-FMK treatment remained 1 year after islet transplantation (Emamaullee *et al.* 2007). Similarly, Pepper and colleagues cultured human islets with a pan-caspase inhibitor (F573) for 24 hours prior to transplantation, then administered F573 for 5 days post transplantation in immunodeficient mice. This approach resulted in improved blood glucose during an intraperitoneal glucose tolerance test (IPGTT) for up to 100 days post transplant and was accompanied by the preservation of β-cell mass and viability (Pepper *et al.* 2017). Other studies using small molecule caspase inhibitors to protect transplanted islets have observed similar results (Emamaullee *et al.* 2008, McCall *et al.* 2011). In addition, several studies found that the blockade of IL-1β and TNFα, classical apoptotic stimuli, significantly improves islet transplant outcomes (Bellin *et al.* 2008, Matsumoto *et al.* 2011, Onaca *et al.* 2020).

These and other studies demonstrate that β-cells undergo apoptosis in response to diabetes-relevant cell death stimuli.

#### Necrosis

β-Cells are also susceptible to non-apoptotic forms of cell death such as necrosis (Hoorens *et al.* 2001, Fehsel *et al.* 2003). Necrosis was first described by Rudolf Virchow in 1858 and is considered distinct from mechanisms of regulated cell death or PCD (Majno & Joris 1995). Necrosis is typically understood as a form of unprogrammed or accidental cell death that results from cellular damage, infection, or trauma (Buja *et al.* 1993). In contrast to apoptosis, necrosis is characterized by lytic loss of membrane integrity that leads to the unregulated release of cell contents (Buja *et al.* 1993, Majno & Joris 1995). Given that these cell contents are not packaged in apoptotic bodies or membrane blebs, this release of cell contents is inflammatory in nature (Davidovich *et al.* 2014, Rock & Kono 2008). Thus, necrosis can be characterized by the release of cellular factors such as high mobility group box 1 (HMGB1), a nuclear-localized protein that is normally associated with chromatin (Raucci *et al.* 2007). In addition, morphological hallmarks of necrotic cell death such as karyolysis, karyorrhexis, and pyknosis, all of which relate to altered morphology of nuclear DNA within the dying cell body, can be used to identify necrotic cells (Anthony 1990). Such changes are not observed in apoptosis, where proteases and nucleases break down nuclear contents into apoptotic bodies (Anthony 1990).

Several studies have found that necrosis contributes to β-cell loss in diabetes. Notably, studies by Fehsel *et al.* examined STZ-treated mouse islets *in vitro* and prediabetic diabetes-prone BB rat pancreas sections *ex vivo* using electron microscopy, quantification of DNA damage, and annexin V staining (Fehsel *et al.* 2003). These studies identified only a small number of cells expressing markers of apoptosis, but β-cells with characteristics of necrosis were significantly more abundant (Fehsel *et al.* 2003). When evaluating mechanisms of IL-1β-mediated cell death in rat β-cells, Steer and colleagues found evidence of necrosis, including loss of viability, a lack of caspase 3 activity, annexin V positivity, and robust HMGB1 release, whereas the apoptosis inducer camptothecin strongly induced caspase 3 activity and annexin V positivity, but not HMGB1 release (Steer *et al.* 2006). In addition, two studies using a rat β-cell line and rat islets found that IL-1β + IFNγ-induced β-cell cytotoxicity was not associated with increased caspase 3 activity or annexin V positivity (Collier *et al.* 2011, 2006). Similarly, TNFα + IFNγ treatment was observed to induce mouse islet cell death in a caspase 3-independent manner (Irawaty *et al.* 2002), and IFNγ + synthetic double-stranded RNA (poly I:C) induces NO-dependent necrosis of rat islet cells (Scarim *et al.* 2001). Necrosis has also been identified as a mediator of β-cell cytotoxicity in islet transplantation, with studies observing characteristics of islet cell necrosis such as pyknotic nuclei and HMGB1 release following islet graft failure (Biarnés *et al.* 2002, Cheng *et al.* 2017, Gebe *et al.* 2018).

Together, these studies provide evidence that β-cells undergo necrosis in response to diabetogenic cell death stimuli.

### Emerging mechanisms of β-cell death

Although necrosis has traditionally been viewed as an unregulated, accidental form of cell death, many of the studies described above identified programmed β-cell death that is morphologically consistent with necrosis. How might this occur when all mechanisms of PCD have customarily been considered apoptosis (Alberts *et al.* 2002, Elmore 2007)? An explanation has surfaced from studies that identified and characterized mechanisms of regulated necrosis which occur downstream of programmed signaling events, are distinct from apoptosis, and constitute novel forms of PCD (Proskuryakov *et al.* 2003, Linkermann & Green 2014, Berghe *et al.* 2014, Conrad *et al.* 2016). These discoveries have been aided by improved definitions of distinct cell death signaling pathways (Galluzzi *et al.* 2009) and have led to renewed interest in understanding molecular mechanisms of PCD and their roles in human disease, including diabetes (Bruni *et al.* 2018, Yang *et al.* 2020, Tonnus *et al.* 2021, Contreras *et al.* 2022). We propose that novel mechanisms of PCD, such as necroptosis, ferroptosis, and pyroptosis, may contribute to T1D pathogenesis, not only as end-stage mechanisms of β-cell loss, but potentially as early-stage mechanisms of islet inflammation and β-cell autoimmunity. In the following section, we review literature on basic mechanisms of regulated necrosis, including necroptosis, ferroptosis, and pyroptosis. We also examine recent studies linking these mechanisms of inflammatory PCD to β-cell cytotoxicity and discuss the need for additional research to examine the relevance of these mechanisms to diabetogenic β-cell death.

#### Necroptosis

**Figure 1:**
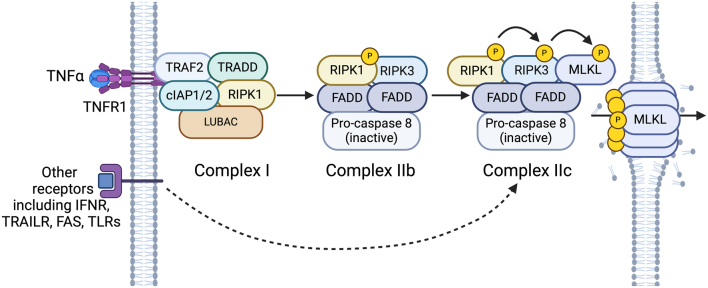
Basic mechanisms of necroptosis signaling. The binding of TNFα to TNFR1 initiates the recruitment of TRAF2, TRADD, RIPK1, cIAP1/2, and LUBAC to form complex I at the receptor. Under appropriate stimulation, RIPK1 associates with pro-caspase 8, FADD, and RIPK3 to form complex IIb. When pro-caspase 8 is inactive, RIPK1 activates RIPK3 via phosphorylation, and activated RIPK3 recruits and phosphorylates MLKL in complex IIc (the necrosome). Phosphorylation of MLKL leads to its conformational change, oligomer formation, membrane translocation, and disruption of membrane integrity, resulting in cell lysis. Other receptors including IFNγR, TRAILR, FAS, and TLRs have also been found to contribute to necrosome formation and necroptosis.

Necroptosis is a regulated form of necrotic cell death that is mediated by receptor-interacting protein kinase 1 (RIPK1), receptor-interacting protein kinase 3 (RIPK3), and mixed lineage kinase domain-like pseudokinase (MLKL) (Linkermann & Green 2014) (Fig. 1). Necroptosis was first identified as a form of cell death that occurs downstream of tumor necrosis factor receptor 1 (TNFR1) signaling when caspase activity is inhibited (Vercammen *et al.* 1998, Liu *et al.* 2003), and the pathway is most well-defined in this context. In canonical necroptosis signaling, TNFα binds to TNFR1, and molecules including TNFR-associated death domain (TRADD), TNFR-associated factor 2 (TRAF2), cellular inhibitors of apoptosis proteins (cIAP1/2), linear ubiquitin chain assembly complex (LUBAC), and RIPK1 are recruited to form complex I (Wilson *et al.* 2009). RIPK1 undergoes extensive ubiquitination and deubiquitination in complex I, and this process regulates its pro-survival and pro-death functions (Brenner *et al.* 2015, Conrad *et al.* 2016, Li *et al.* 2020). In its pro-survival role, linear ubiquitination of RIPK1 in complex I is crucial for NF-κB activation, leading to the upregulation of pro-survival gene expression (Gerlach *et al.* 2011, Berghe *et al.* 2014). Deubiquitination of RIPK1 facilitates the formation of complex II with caspase-8 and Fas-associated death domain (FADD)(Brenner *et al.* 2015). When caspases are active, caspase 8 in complex IIa or IIb inactivates RIPK1 (Lin *et al.* 1999) and RIPK3 (Feng *et al.* 2007), eliciting caspase 3/7 activation and apoptosis (Brenner *et al.* 2015). When caspases are inactive, however, complex IIc (the necrosome) forms via phosphorylation and interaction of RIPK1 and RIPK3, leading to the recruitment and phosphorylation of MLKL, the terminal effector of necroptosis (Cai *et al.* 2014, Dondelinger *et al.* 2014). This RIPK1–RIPK3–MLKL phosphorylation cascade leads to a conformational change in MLKL that exposes its four-helix bundle domain and leads to its oligomerization, membrane translocation, and loss of cell membrane integrity (Hildebrand *et al.* 2014). Although the precise mechanisms of necroptosis execution downstream of MLKL oligomerization are not fully established, membrane-localized MLKL pore formation results in cell swelling and lysis (Cai *et al.* 2014). This lysis releases cell contents including danger-associated molecular patterns (DAMPs) that promote inflammation and immune responses (Kaczmarek *et al.* 2013, Murai *et al.* 2018). In addition to TNFR1, several other receptors including interferon-gamma receptor (IFNGR), toll-like receptors (TLRs), Fas receptor, and TNF-related apoptosis-inducing ligand receptor (TRAILR) have been implicated in necroptosis signaling and are thought to converge at the level of necrosome formation (Holler *et al.* 2000, Meurette *et al.* 2005, Thapa *et al.* 2013, Linkermann & Green 2014, Berghe *et al.* 2014).

Stimuli known to induce necroptosis have previously been observed to elicit caspase-independent β-cell death. For example, TNFα + IFNγ (Irawaty *et al.* 2002), IL-1β + IFNγ (Collier *et al.* 2006, 2011), and IFNγ + double stranded RNA (Scarim *et al.* 2001) have been found to induce β-cell death with necrotic morphology in the absence of caspase activation, consistent with regulated necrosis. Although these studies did not examine whether such mechanisms of β-cell death are biochemically consistent with necroptosis, they provide early evidence that diabetes-relevant stimuli can trigger programmed β-cell death distinct from caspase-mediated apoptosis. In addition, recent biochemical and transcriptomics studies have identified RIPK1, RIPK3, and MLKL expression in rodent and human β-cells (Thompson *et al.* 2019, Yang *et al.* 2020, Contreras *et al.* 2022, Elgamal *et al.* 2023), again rationalizing the need for additional research into the role of necroptosis signaling in β-cell cytotoxicity.

RIPK1 is a key upstream regulator of necroptosis. It is a multifunctional protein with an N-terminal kinase domain, a C-terminal death domain, and a receptor-interacting protein (RIP) homotypic interaction motif (RHIM) that mediates interactions with RIPK3 and other RHIM-containing molecules (Li & Yuan 2023). Human Pancreas Analysis Program (HPAP) data indicate that Ripk1 gene expression is increased 2.7-fold in β-cells from individuals with T1D compared to those without diabetes (Kaestner *et al.* 2019), and several studies have investigated the role of RIPK1 kinase function in β-cell cytotoxicity using small molecule RIPK1 kinase inhibitors. One study evaluated the effects of necrostatin-1 (Nec-1) on human islets cultured in low-nutrient and low-oxygen conditions, finding that Nec-1 treatment reduced dsDNA and uric acid release from human islets, indicative of decreased lytic cell death (Paredes-Juarez *et al.* 2015). Another study in rodent β-cell lines and mouse islets found that inhibition of RIPK1 kinase function with Nec-1 protects from NO donor-induced β-cell cytotoxicity *in vitro* (Tamura *et al.* 2011). In a study of young porcine islets, Nec-1 was found to improve islet viability in response to NO or hypoxia in a dose-dependent manner *in vitro* (Lau *et al.* 2020) and another recent study found that Nec-1 treatment prevented β-cell loss in a zebrafish model of overnutrition and insulin resistance (Yang *et al.* 2020). Our recent study found that CRISPR-mediated Ripk1 gene editing protects NIT-1 β-cells from TNFα-induced cell death both in the presence and absence of caspase 3/7 activation (Contreras *et al.* 2022). In contrast to these studies, mice harboring a Ripk1^S25D/S25D^ mutation that mimics inhibitory phosphorylation of RIPK1 were not protected from hyperglycemia following STZ or HFD (Takiishi *et al.* 2023). Considering that RIPK1 has several inhibitory and activating phosphorylation sites as well as actions in multiple tissues (Li & Yuan 2023), studies evaluating Ripk1 kinase dead (Ripk1^D138N/D138N^) or Ripk1 tissue-specific knockout (Ripk1^flox/flox^) mice may shed additional light on the role of RIPK1 in diabetogenic β-cell decline *in vivo*. In sum, these data indicate that RIPK1 plays a key role in regulating β-cell fate and suggest that inhibition of RIPK1 kinase activity could protect β-cells from diabetogenic insults.

RIPK3 is a major downstream phosphorylation target of RIPK1 that is known to promote inflammation and cell death signaling in non-islet cell types (Newton *et al.* 2014, Lawlor *et al.* 2015, Orozco & Oberst 2017). In comparison to RIPK1, RIPK3 retains an N-terminal kinase domain and a RIP homotypic interaction motif (RHIM) but does not contain a death domain (Moriwaki & Chan 2017). Human Pancreas Analysis Program (HPAP) data indicate that Ripk3 gene expression is increased 2.2-fold in β-cells from individuals with T1D compared to those without (Kaestner *et al.* 2019), and recent studies have begun to decipher the roles of RIPK3 in β-cell inflammation and cell death signaling. Our recent observations revealed that β-cell lines and isolated mouse islets express RIPK3 at levels similar to other necroptosis-susceptible cells (Contreras *et al.* 2022). This work also revealed that CRISPR-mediated gene editing of Ripk3 protects NIT-1 β-cells from TNFα-induced cell death independent of caspase 3/7 activation *in vitro,* and that RIPK3 knockout mice are protected from STZ-induced hyperglycemia *in vivo* (Contreras *et al.* 2022). These data suggest that TNFα elicits both caspase-mediated apoptosis and RIPK3-mediated necroptosis in NIT-1 β-cells, with necroptosis serving as a secondary form of cell death when caspase activation is insufficient to elicit apoptosis.

RIPK3 also promotes inflammation in a cell death-independent manner in non-islet cell types (Orozco & Oberst 2017), and a recent study found that RIPK3 contributes to inflammation in zebrafish, mouse, and human β-cells (Yang *et al.* 2020). This work found that endoplasmic reticulum (ER) stress activates RIPK3 to elicit NF-κB-mediated proinflammatory gene expression in cultured β-cells and that RIPK3 kinase inhibition protects mouse islets from palmitate-induced β-cell dysfunction *in vitro* (Yang *et al.* 2020). Islet inflammation, β-cell dysfunction, and β-cell loss were found to occur in a RIPK3-dependent manner in a zebrafish model of β-cell cytotoxicity, and human islets grafted in hyperglycemic mice underwent a marked increase in RIPK3 and NF-κB activation that was accompanied by increased islet macrophage infiltration (Yang *et al.* 2020). Moreover, RIPK3 was found to promote amyloid-associated islet inflammation and β-cell cytotoxicity in a humanized mouse model of islet amyloidosis (Mukherjee *et al.* 2024). Although further investigation is needed to clarify the specific roles of RIPK3 in islet inflammation and β-cell death, these data indicate that therapies targeting RIPK3 could protect β-cells from diabetes-relevant cytotoxic stimuli.

Necroptosis has also been identified as a mediator of graft failure following transplantation, including in the setting of heart, kidney, and lung transplants (Lau *et al.* 2013, Pavlosky *et al.* 2014, Kim *et al.,* 2018). In a study of islet transplantation, porcine islets treated with the RIPK1 kinase inhibitor Nec-1 were found to exhibit increased insulin content and insulin secretion following glucose stimulation *in vitro*, and treatment of islets with Nec-1 prior to transplantation in diabetic athymic mice resulted in shorter times to normoglycemia and higher plasma insulin levels *in vivo* (Lau *et al.,* 2021). In contrast, a study evaluating transplantation of RIPK3 deficient mouse islets found them to be normally susceptible to CD4+ T cell-mediated destruction *in vivo* (Zhao *et al.,* 2015). Additional studies are warranted to understand the role of necroptosis in islet graft failure.

In sum, the literature reviewed here indicates that necroptosis signaling components such as RIPK1 and RIPK3 play important roles in regulating β-cell fate. However, additional *in vitro* and *in vivo* studies are needed to understand the mechanisms of β-cell necroptosis and its relevance to diabetes pathogenesis.

#### Ferroptosis

**Figure 2:**
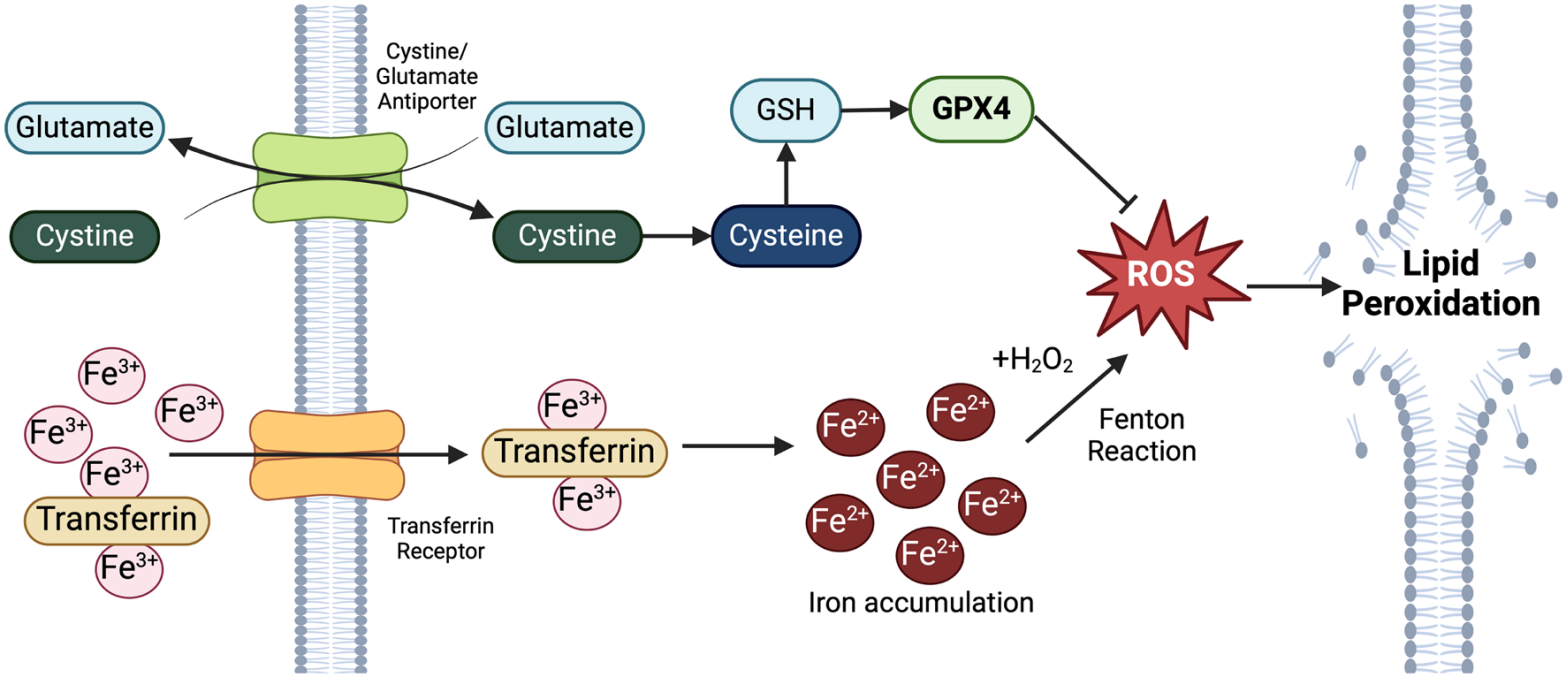
Basic mechanisms of ferroptosis signaling. Ferroptosis is triggered by an imbalance of intracellular free iron and cell antioxidant capacity, eventually leading to excessive lipid peroxidation and cell lysis. Cystine is essential for glutathione (GSH) production, and GSH is a cofactor for glutathione peroxidase 4 (GPX4), a critical lipid peroxidase. Iron is taken up by the cell, and stable Fe^3+^ can be converted to free redox-active Fe^2+^. This transition facilitates the accumulation of reactive oxygen species (ROS) through the Fenton reaction with H_2_O_2_, and GPX4 is needed to counteract lipid peroxidation that arises through this process. Stimuli that increase intracellular free iron, reduce cellular cystine uptake, or decrease cellular antioxidant capacity can lead to ferroptosis.

Ferroptosis is a non-apoptotic form of PCD linked to lethal lipid peroxidation from iron-dependent ROS accumulation (Xie *et al.* 2016) (Fig. 2). It is identified morphologically by a unique intact nucleus, reduced mitochondrial volume, increased lipid bilayer membrane density and rupture, and reduced mitochondrial cristae (Xie *et al.* 2016). Ferroptosis was identified in 2012 when Dixon *et al.* studied the mechanisms and morphology of cell death following treatment of fibrosarcoma cells with erastin (Dixon *et al.* 2012), a small molecule that prevents the synthesis of the antioxidant glutathione via inhibition of the cystine–glutamate antiporter system X_C_^–^ and reduced intracellular cysteine (Dixon *et al.* 2012, Sun *et al.* 2018). Cysteine is a rate-limiting substrate for the biosynthesis of glutathione (GSH), and GSH is a cofactor of glutathione peroxidase 4 (GPX4), a major lipid peroxide scavenger (Brigelius-Flohé & Maiorino 2013). Erastin itself was discovered by Dolma and colleagues while screening for compounds that could kill human tumor cells and was found to be lethal to tumor cells with small T oncoproteins and oncogenic alleles, although the dead cells did not exhibit hallmarks of apoptosis such as chromatin condensation or DNA fragmentation (Dolma *et al.* 2003). In line with these findings, Yang *et al.* found that erastin treatment decreased NADPH oxidation and GPX4 activity in human foreskin fibroblasts, consistent with cytotoxic lipid peroxidation (Yang *et al.* 2014). Moreover, inhibition of GPX4 activity, increased ROS generation, and increased lipid peroxidation were identified as common mechanisms elicited by several ferroptosis-inducing small molecules including erastin and RSL3, a ferroptosis inducer and potent inhibitor of GPX4 (Yang *et al.* 2014, Sui *et al.* 2018). In sum, the absence of sufficient GPX4 activity leads to iron-dependent ROS production, lipid peroxidation, and disruption of plasma membrane integrity, leading to ferroptotic cell death (Bruni *et al.* 2018).

Studies to understand the role of ferroptosis in β-cell death are now emerging. β-cells naturally express low levels of antioxidant enzymes compared to other hormone-secreting cells (Grankvist *et al.* 1981, Lei & Vatamaniuk 2011), leaving them vulnerable to oxidative stress and, potentially, ferroptosis. Following treatment with erastin or RLS3, human islets exhibited decreased glucose-stimulated insulin release and viability (as measured by LDH release), indicating they are susceptible to ferroptosis (Bruni *et al.* 2018). Moreover, treatment with ferrostatin (Fer-1, a ferroptosis inhibitor) for 24 hours protected human islets from erastin-induced impairments in insulin secretion and viability (Bruni *et al.* 2018, Miotto *et al.* 2020). Another recent study evaluated the role of ferroptosis in high glucose-, proinflammatory cytokine-, hydrogen peroxide (H_2_O_2_)- and STZ-induced β-cell death *in vitro* (Stancic *et al.* 2022). The authors found that each of these stimuli increased cell death in RIN-5F rat insulinoma cells, and that this cytotoxicity occurred in conjunction with increased abundance of ROS, lipid peroxides, and iron as well as decreased GPX4 expression (Stancic *et al.* 2022). Cotreatment with Fer-1 rescued RIN-5F cell death caused by high glucose, H_2_O_2_, or STZ but failed to protect from proinflammatory cytokine-induced death (Stancic *et al.* 2022). With respect to *in vivo* studies, STZ treatment was found to increase islet lipid peroxidation and elicit β-cell loss in C57BL/6 mice, and these effects were associated with downregulation of GPX4 and NRF2, a transcription factor that regulates expression of antioxidant genes (Markelic *et al.* 2023). Treatment with Fer-1 was found to reduce lipid peroxidation, upregulate GPX4 and NRF2, and ameliorate β-cell loss in STZ-treated mice (Markelic *et al.* 2023). A separate study investigated the role of cystine import in β-cell cytotoxicity using a mouse model deficient for Slc7a11, a cystine/glutamate antiporter needed for GSH production and GPX4 activity (de Baat *et al.* 2023). Loss of *Slc7a11* reduced levels of cystine and glutathione in mouse islets and led to reduced insulin secretion, downregulation of β-cell identity genes, and an increase in ER stress markers (de Baat *et al.* 2023). These observations appeared to be dependent on β-cell phenotypes, as myeloid-specific deletion of *Slc7a11* did not elicit such changes (de Baat *et al.* 2023).

Iron homeostasis also plays a critical role in ferroptosis. Transferrin and transferrin receptors transport iron (Fe^3+^) into the cytosol for storage and controlled release by ferritin in a process that regulates iron balance (Plays *et al.* 2021, Ems *et al.* 2023). Excess free iron (Fe^2+^) reacts with hydrogen peroxide through the Fenton reaction, generating toxic hydroxyl radicals that can attack the lipid membrane (Xie *et al.* 2016), and several studies have linked elevated iron concentrations to β-cell cytotoxicity. Therefore, understanding β-cell iron homeostasis could lead to a greater appreciation of the mechanisms that elicit ferroptosis in β-cells. In 1994, a mouse model of hemochromatosis (*Hfe* ^−/−^) was examined and found to have a 72% increase in islet iron content compared with wild-type mice, and this was associated with reduced *Ins1* and *Ins2* expression and diminished insulin content *in vitro* (Cooksey *et al.* 2004). At 6-8 months of age, *Hfe* ^−/−^ mice exhibited reduced islet size, increased islet caspase 3 activity, and increased islet TUNEL staining compared to wild-type mice (Cooksey *et al.* 2004). Masuda *et al.* found that exposure of rat islets to iron sucrose led to oxidative stress and pancreatic islet cell death in a dose-dependent manner (Masuda *et al.* 2013), and another study found that treatment of MIN6 cells with high iron concentrations resulted in a 15-fold increase in cellular iron content, elevated levels of lipid peroxidation, decreased insulin content and secretion, and reduced viability (Blesia *et al.* 2021).

The role of ferroptosis in islet transplantation has also been an area of research interest, but its role in islet graft failure remains unclear. Although Bruni *et al.* found that human islets are susceptible to ferroptosis *in vitro*, neither chemical induction (with erastin) nor inhibition (with Fer-1) of ferroptosis altered human islet engraftment following transplant in immunodeficient mice (Bruni *et al.,* 2018). However, an early study found that desferrioxamine (DFO, an iron chelator) prevented islet allograft damage (Bradley *et al.,* 1986). Although the mechanisms underlying this protection were not determined, the results are suggestive of a role of ferroptosis (Bradley *et al.,* 1986). In more recent studies, encapsulated human islets pretreated with DFO were found to have increased insulin release following transplant in STZ-treated mice (Vaithilingam *et al.,* 2010), and bilirubin was found to decrease islet graft ferroptosis via effects on GPX4 expression, NRF2 expression, and iron chelation (Yao *et al.,* 2020).

Together, these studies have advanced our understanding of β-cell ferroptosis. However, additional studies using physiologically relevant models of diabetes are needed to clarify the importance of β-cell ferroptosis in diabetes pathogenesis.

#### Pyroptosis

**Figure 3:**
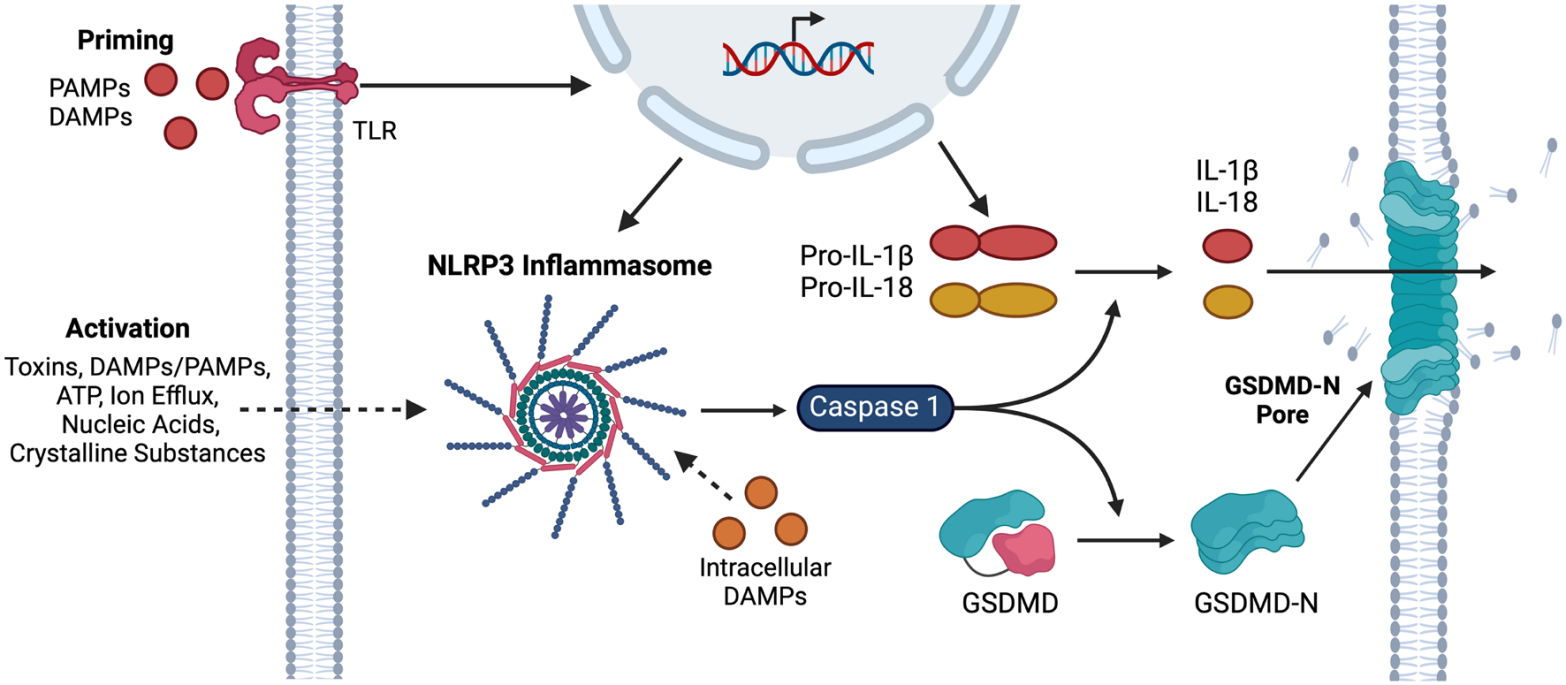
Basic mechanisms of canonical pyroptosis signaling. In canonical pyroptosis, extracellular signals such as danger-associated molecular patterns (DAMPs) and pathogen-associated molecular patterns (PAMPs) elicit priming via upregulation of the NLRP3 inflammasome, pro-IL-1β, and pro-IL-18. During the activation phase, stimuli trigger the NLRP3 inflammasome leading to caspase 1 activation. Caspase 1 then cleaves gasdermin D (GSDMD), resulting in the formation of GSDMD-N, which forms pores in the cell membrane. Simultaneously, caspase 1 generates mature IL-1β and IL-18, which are released through GSDMD-N pores, culminating in pyroptosis.

Pyroptosis is an inflammatory form of lytic PCD that occurs in response to infection and generates an immune response (Bergsbaken *et al.* 2009). A central molecular player in pyroptosis is gasdermin D (GSDMD), a protein that contains an N-terminal pore-forming domain and a C-terminal repressor domain separated by a linker region (Cookson & Brennan 2001). Activation of inflammatory caspases (caspase 1, 4, 5, and 11) elicits cleavage of the GSDMD linker domain, releasing N-terminal GSDMD (GSDMD-N) that facilitates membrane pore formation, allowing release of inflammatory molecules including HMGB1 and IL-1β, and promoting cell death (Kuang *et al.* 2017, Volchuk *et al.* 2020, Phulphagar *et al.* 2021) (Fig. 3). Pyroptosis has been studied in the context of immune responses to infection (Lara-Tejero *et al.* 2006) and inflammatory disease pathogenesis (Wu *et al.* 2022). Like apoptosis, pyroptosis exhibits DNA damage, chromatin condensation, and formation of membrane blebs (Bergsbaken *et al.* 2009). However, pyroptosis is a distinct form of lytic cell death associated with membrane leakage, flattening of the cytoplasm, and inflammation (Chen *et al.* 2016).

In canonical pyroptosis signaling, PAMPs and DAMPs are recognized extracellularly by pattern-recognition receptors (PRRs) such as TLRs. In this priming phase of pyroptosis, extracellular stimulation of PRRs upregulates the expression of NLR family pyrin domain containing 3 (NLRP3) and cytokines in the cell. In the activation phase, cytosolic PRRs such as NOD-like receptors (NLRs) are activated by stimuli such as ATP, nucleic acids, crystalline substances, and intracellular DAMPs and PAMPs, leading to NLR-directed assembly of the NLRP3 inflammasome (Song *et al.* 2022). The relationship between caspase 1 activity and pyroptosis was uncovered when *Salmonella*-induced cell death was found to be blocked by a small molecule caspase 1 inhibitor (Brennan & Cookson 2000). It was later revealed that a complex including caspase 1, caspase 5, PYCARD, and NALP1 controls the activation of inflammatory caspases, and this complex was termed the inflammasome (Martinon *et al.* 2002). Depletion of PYCARD or NALP1 was found to decrease caspase 1 and caspase 5 activation and to reduce pro-IL-1β processing (Martinon *et al.* 2002). Agostini and colleagues showed that NLRP3 contributes to inflammasome assembly, with NLRP3 associating with PYCARD, CARD8, and caspase 1 to generate an inflammasome complex with high pro-caspase 1 and pro-IL-1β processing activity (Agostini *et al.* 2004). Activated caspase 1 cleaves GSDMD, promoting GSDMD-N pore formation in the plasma membrane (Bergsbaken *et al.* 2009). The importance of GSDMD in pyroptosis was identified using CRISPR screens (Shi *et al.* 2015) and GSDMD-null mice (Russo *et al.* 2016). In both cases, *Gsdmd* deficient BMDMs exhibited reduced IL-1β release and cytolysis following treatment with known inducers of pyroptosis (Shi *et al.* 2015, Russo *et al.* 2016). Once GSDMD-N pores are formed on the plasma membrane, inflammatory mediators including HMGB1, IL-1β, and IL-18 are released to elicit immune responses (Fink & Cookson 2006, Russo *et al.* 2016).

The growing understanding of pyroptosis has led to several studies of this form of cell death in β-cells. Human Pancreas Analysis Program (HPAP) data indicates that GSDMD gene expression is increased 2.2-fold in β-cells from individuals with T1D compared to non-diabetic individuals (Kaestner *et al.* 2019), suggesting a role for GSDMD in β-cell cytotoxicity in this disease. In line with these findings, a recent study found that GSDMD is upregulated at both the RNA and protein levels in response to proinflammatory cytokines (IL-1β+IFNγ+TNFα) in human β-cells (EndoC-βH5) and human islets (Frørup *et al.* 2024). In addition, a 2021 study investigating the effect of miR-17-5p on β-cell cytotoxicity found that miR-17-5p improved glucose homeostasis in C57B/L6 mice subjected to HFD and STZ in vivo, and this phenotype was associated with increased pancreas insulin area, decreased caspase 1 activation, and reduced GSDMD-N expression ( Liu *et al.* 2021*b*). Irisin, a hormone released from skeletal muscle that mediates beneficial effects of exercise (Vaughan *et al.* 2014), was found to reduce caspase 1 activity as well as GSDMD-N, IL-1β, and IL-18 protein expression, indicative of protection from pyroptosis signaling (Tan *et al.* 2023). Other molecules, including the natural products salidroside and emodin, have also been shown to decrease GSDMD expression in INS-1 cells following exposure to high glucose (Xing *et al.* 2022, Zhou *et al.* 2023), and the sodium/glucose cotransporter 2 inhibitor (SGLT2i) empagliflozin was shown to reduce NLRP3, caspase 1, and GSDMD expression in mouse β-cells (βTC-6) in response to high glucose, an effect that was also observed in islets of empagliflozin-treated *db/db* mice (Liu *et al.* 2021*a*).

Although direct links between pyroptosis and islet graft failure have yet to be established, evidence indicates pyroptosis-relevant molecules such as the NLRP3 inflammasome and IL-1β are involved in islet transplant demise. For example, rat islets transplanted under the kidney capsule of immunodeficient mice exhibited significant increases in NLRP3 and IL-1β expression 2 days post transplant (Lavallard *et al.* 2020), and transplantation of either Nlrp3^−/−^ or Casp1^−/−^ islets in STZ-treated mice accelerated the restoration of normoglycemia and improved glucose tolerance compared to WT islets (Wrublewsky *et al.* 2022).

Together, these studies suggest that pyroptosis contributes to diabetes-relevant β-cell cytotoxicity. Additional studies in disease-relevant models are needed to understand the importance of pyroptosis in diabetogenic β-cell demise.

### Heterogeneity of β-cell death responses

In this review, we’ve examined studies of established and emerging mechanisms of cell death and their relevance to β-cell cytotoxicity. Although one may assume that a cell death stimulus elicits a single mode of death, evidence indicates that cell death responses are heterogeneous within a cell population. Indeed, several studies have observed that a specific cell death stimulus induces both apoptosis and necrosis concurrently within a population of cells. For example, glutamate was found to elicit early necrosis in a subset of neurons (characterized by nuclear swelling and release of cellular debris), and cells spared from early necrosis later underwent apoptosis, as evidenced by the formation of apoptotic bodies and chromatin degradation (Ankarcrona *et al.* 1995). Acetaminophen overdose causes cell death in mouse hepatocytes with hallmarks of both necrosis and apoptosis including TUNEL positivity, membrane permeabilization, and caspase 3 activation (Kon *et al.* 2004). Tanshinone IIA, a natural product of *Salvia miltiorrhiza*, was found to simultaneously elicit apoptosis and necroptosis in HepG2 cells, and this apoptosis could be converted to necroptosis via treatment with a small molecule pan-caspase inhibitor (Lin *et al.* 2016). Preedy and colleagues recently observed heterogeneity in TNFα/TNFR1 signaling within a population of mouse fibroblasts, leading them to advocate for the use of novel live-cell imaging techniques to understand cell fate at the level of single cells (Preedy *et al.* 2024). Such heterogeneity in cell death responses may be influenced by different transcriptional or mitochondrial states of single cells at the time of exposure to a cell death stimulus.

Heterogeneity of cell death responses has also been observed in β-cells. For example, Saini *et al.* found that STZ treatment elicited both apoptosis and necrosis in INS-1 cells *in vitro*, with necrosis being more common than apoptosis at high STZ concentrations (Saini *et al.* 1996). β-cells isolated from Wistar rats were found to undergo both necrosis and apoptosis following oleate and palmitate treatment, as determined by neutral red and PI staining (Cnop *et al.* 2001). Saldeen and colleagues showed that treatment with IL-1β, IFNγ, and TNFα increased both necrosis (17% of cells) and apoptosis (5% of cells) in isolated rat islets via a Bcl-2-inhibitable pathway (Saldeen 2000). Moreover, IFNγ and double-stranded RNA (Poly I:C) treatment was found to stimulate a five-fold increase in necrosis and a seven-fold increase in apoptosis after 48 hours in Sprague-Dawley rat islets, as determined by acridine orange and ethidium bromide staining (Scarim *et al.* 2001). In addition, islet graft failure was found to be associated with both apoptosis and necrosis following transplantation in STZ-treated C57BL/6 mice (Biarnés *et al.* 2002). Although transcriptional and functional heterogeneity within β-cell populations is well recognized, these findings indicate that heterogeneity in the context of β-cell death deserves further consideration.

**Figure 4:**
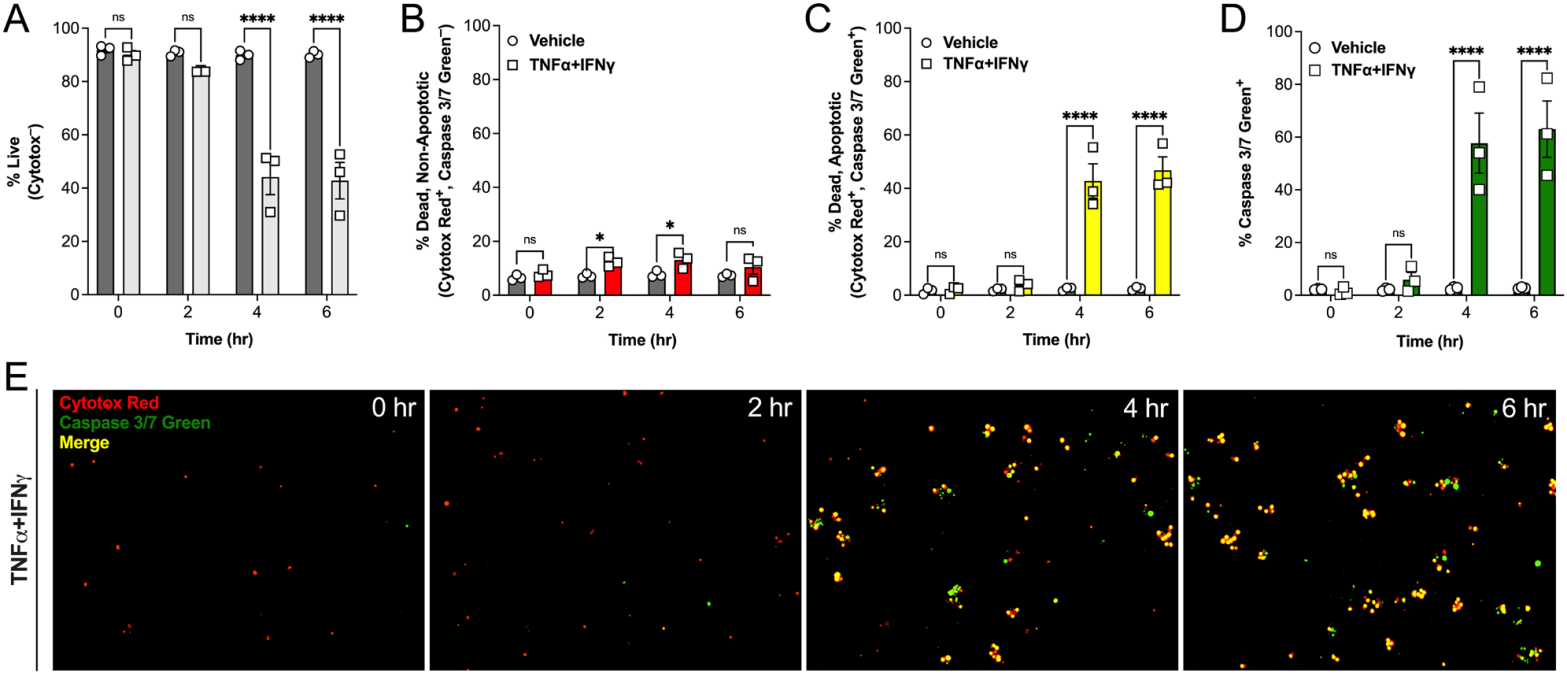
Heterogeneity of β-cell death responses. A Sartorius Incucyte S3 live cell imaging and analysis instrument was used to monitor cell death (Cytotox Red, Sartorius, 250 nM) and caspase 3/7 activation in real-time (Caspase 3/7 Green, Sartorius, 5 µM). NIT-1 β-cells were plated and treated with vehicle (circles) or TNFα (40 µg/mL) + IFNγ (100 µg/mL) (squares). Cytotox Red−, Caspase 3/7 Green+ (caspase 3/7 activation), Cytotox Red+, Caspase 3/7 Green− (dead, non-apoptotic), and Cytotox Red+; Caspase 3/7 Green+ (dead, apoptotic) objects were monitored hourly over 6 h and quantified at 0, 2, 4, and 6 h post treatment. Total cell count was determined using AI-mediated cell-by-cell phase contrast analysis, and data is represented as a percent of total cells. (A) Percent live cells (dark gray: vehicle, light gray: TNFα+IFNγ), (B) percent non-apoptotic dead cells (Cytotox Red+, Caspase 3/7 Green−; gray: vehicle, red: TNFα+IFNγ), (C) percent apoptotic dead cells (Cytotox Red+; Caspase 3/7 Green+; gray: vehicle, yellow: TNFα+IFNγ), and (D) percent caspase 3/7 activated live cells (Cytotox Red−, Caspase 3/7 Green+; gray: vehicle, green: TNFα+IFNγ) were quantified. (E) Representative images of Cytotox Red and Caspase 3/7 Green-positive NIT-1 cells 0, 2, 4, and 6 h post TNFα+IFNγ treatment (10× magnification). Data are presented as mean ± s.e.m . and were analyzed by two-way ANOVA with Holm–Sidák multiple comparisons correction. ns, not significant; *P < 0.05; ****P < 0.0001, *P* > 0.05.

We performed studies to quantify apoptotic and non-apoptotic cell death in NIT-1 β-cells derived from the NOD mouse model of T1D at the single-cell level. NIT-1 β-cells are known to be sensitive to TNFα- and IFNγ-induced cytotoxicity (Hamaguchi *et al.* 1991, Stephens *et al.* 1999, Contreras *et al.* 2022), and this has been attributed primarily to apoptosis. Here, we treated NIT-1 β-cells with TNFα+IFNγ and evaluated cell death using Cytotox Red Dye, a membrane-impermeable DNA binding dye that enters cells with the loss of membrane integrity, and we concurrently monitored caspase 3/7 activation using Caspase 3/7 Green Dye, a membrane-permeable dye that is non-fluorescent until cleaved by caspase 3 or 7, leading to the release of the DNA binding dye and nuclear staining (Fig. 4). This approach allows for hourly quantification of Cytotox Red-positive cells, Caspase 3/7 Green-positive cells, and cells positive for both at the single-cell level. Consistent with previous findings, we found that TNFα + IFNγ treatment provokes early non-apoptotic cell death (Fig. 4B and E, Cytotox Red+, Caspase 3/7 Green−, 2–4 h) followed by the induction of caspase 3/7 activation and apoptotic cell death (Fig. 4C–E, Cytotox Red+, Caspase 3/7 Green+, 4–6 h) in NIT-1 β-cells. We believe the use of novel methodologies such as these will enable a greater understanding of the mechanisms that underlie diabetogenic β-cell death.

## Characterization of distinct mechanisms of PCD

Given the variety of cell death signaling mechanisms that β-cells may employ, methodologies to identify specific forms of cell death are needed. β-cell death has often been labeled as apoptosis without attendant evidence of biochemical or morphological markers of apoptosis such as caspase activation, DNA laddering, or the presence of apoptotic bodies. In recent years, improved understanding of the processes underlying cell death and advances in methods to monitor it have allowed more specific characterization of mechanisms thereof. In light of this growing understanding, guidelines for appropriate classification of various forms of cell death have been established by the Nomenclature Committee on Cell Death (Kroemer *et al.* 2009). With the publication of “Classification of cell death: recommendations of the Nomenclature Committee on Cell Death” (Kroemer *et al.* 2009), “Guidelines for the use and interpretation of assays for monitoring cell death in higher eukaryotes” (Galluzzi *et al.* 2009) and subsequent updates (Galluzzi *et al.* 2012, 2018) the Committee has provided detailed information on biochemical, morphological, and functional characterization of cell death, and we suggest interested readers refer directly to these works.

Characterizing the specific mode of death a cell undergoes requires a high degree of specificity. Several DNA-binding dyes are used to identify dead cells, and they are often described as specifically detecting apoptosis. For example, TUNEL is commonly marketed as an apoptosis-specific marker and is widely employed to identify apoptotic cells (Butler *et al.* 2003, Meier *et al.* 2005, Jurgens *et al.* 2011). However, studies indicate that TUNEL staining identifies dead cells of both apoptotic and necrotic origin (Grasl-Kraupp *et al.* 1995, Kelly *et al.* 2003), and the DNA fragmentation identified with TUNEL is a known feature of various forms of cell death (Higuchi 2003). Given that altered membrane composition is also a common feature of many forms of PCD, methods to identify dead cells that rely on changes in membrane integrity, including DNA-binding dyes such as SYTOX (De Schutter *et al.* 2021) and propidium iodide (PI) (Kelly *et al.* 2003) and phosphatidylserine detection with annexin V (Crowley *et al.* 2016), have similar limitations in their ability to discriminate between distinct mechanisms of cell death. Therefore, the use of multiple methods to characterize cell death phenotypes is helpful in distinguishing between distinct forms of cell death. For example, luminogenic and fluorogenic caspase substrates can be employed to monitor caspase activity in either static or real-time assays (Shi *et al.* 2012), and genetically encoded caspase activity biosensors have also been developed (Zhang *et al.* 2013). As described above, morphological characterization of dead or dying cells (e.g., identification of apoptotic bodies, organelle swelling, or plasma membrane rupture) can also be employed to differentiate between mechanisms of cell death. Specific biochemical characteristics of apoptosis, necroptosis, ferroptosis, and pyroptosis have been described, and identification of these biochemical signatures can be used to distinguish these forms of cell death. In addition, methodologies that allow single cell analysis of cell death responses enable a better understanding of the mechanisms of cell death that arise in response to a specific stimulus. We have provided a brief overview of cell death pathways, their morphological features, and methods of detection in Table 2. The selection presented in Table 2 reflects methodologies discussed within the context of this review. In addition, “Guidelines for the use and interpretation of assays for monitoring cell death in higher eukaryotes” provides a more comprehensive classification of distinct forms of cell death and describes tools to accurately characterize mechanisms of cell death (Galluzzi *et al.* 2009, 2018). We believe a more robust understanding of β-cell death signaling pathways will accelerate efforts to identify β-cell protective therapeutics for use in diabetes.

**Table 2:** Morphological features, detection techniques, and biomarkers of cell death.

Cell death mechanism	Morphology	Detection techniques
Apoptosis	Cytoplasmic shrinkage; nuclear fragmentation; plasma membrane blebbing; chromatin condensation; DNA fragmentation	Caspase 3/7 activation; DNA laddering
Necrosis	Organelle swelling; plasma membrane rupture; karyolysis; karyorrhexis; pyknosis	HMBG1 release,^a^ LDH release^a^
Necroptosis	Plasma membrane rupture, intracellular leakage, cell swelling, and an intact nucleus	RIPK1, RIPK3, MLKL phosphorylation
Ferroptosis	Plasma membrane rupture; intracellular leakage; reduced mitochondrial volume and cristae; intact nucleus	Fe^2+^ release; GPX4, GSH expression; lipid peroxidation detection
Pyroptosis	Membrane pore formation; plasma membrane blebbing, intracellular leakage; cytoplasm flattening; chromatin condensation; DNA damage	Caspase 1 activation; cleavage of GSDMD; IL-1β, IL-18 release

^a^Techniques that can be applied to other forms of lytic cell death.

## Conclusion

β-Cell death is an important contributor to β-cell loss, insulin insufficiency, and hyperglycemia in T1D. Several factors may trigger β-cell cytotoxicity in the pathogenesis of T1D, including metabolic stress, ER stress, inflammation, oxidative stress, and autoimmune attack. Given that identification of individuals at risk for T1D prior to the clinical onset of the disease is now possible, it is imperative that we identify novel therapeutics to protect β-cells in these individuals. To do so, we must better understand the multitude of signaling pathways that underlie programmed β-cell death, how these pathways are triggered in the pathogenesis of T1D, and how they relate to the loss of β-cell mass and the induction of islet inflammation and β-cell autoimmunity over time. Given the current evidence for novel forms of PCD such as necroptosis, ferroptosis, and pyroptosis in β-cells, we propose that such mechanisms may contribute to T1D pathogenesis both as early-stage mechanisms that elicit islet inflammation and β-cell autoimmunity and as late-stage mechanisms of β-cell loss. However, additional studies in disease-relevant models are needed to establish the importance of these forms of cell death to β-cell demise in T1D. Unraveling the pathways involved in β-cell death signaling more clearly may lead to novel approaches to prevent β-cell death, promote β-cell survival, and maintain insulin production and glucose homeostasis in individuals with T1D.

## Declaration of interest

The authors declare that there is no conflict of interest that could be perceived as prejudicing the impartiality of the research reported.

## Funding

This work was supported in part by funding from the U.S. Department of Veterans Affairshttp://dx.doi.org/10.13039/100000738 (IK2 BX004659 to ATT), the Richard L. Roudebush VA Medical Center, the National Institutes of Healthhttp://dx.doi.org/10.13039/100000002 (P30 DK097512 to Indiana Universityhttp://dx.doi.org/10.13039/100006733 Center for Diabetes and Metabolic Diseases, and T32 DK064466 to Indiana Universityhttp://dx.doi.org/10.13039/100006733 Diabetes and Obesity Research Training Program), and the Ralph W. and Grace M. Showalter Research Trust (080657-00002B to ATT).

## Author contribution statement

K.C., N.M., and C.J.C. performed research, analyzed and interpreted literature, and wrote the manuscript. A.T.T. performed research, analyzed and interpreted literature, and wrote and revised the manuscript.
